# Fecal Microbiota Transplantation Ameliorates Active Ulcerative Colitis by Downregulating Pro-inflammatory Cytokines in Mucosa and Serum

**DOI:** 10.3389/fmicb.2022.818111

**Published:** 2022-04-04

**Authors:** Wen-Hui Zhang, Ze-Yu Jin, Zhong-Hua Yang, Jia-Yi Zhang, Xiao-Han Ma, Jing Guan, Bao-Lin Sun, Xi Chen

**Affiliations:** ^1^Department of Gastroenterology, The First Affiliated Hospital of Anhui Medical University, Hefei, China; ^2^USTC-IAT and Chorain Health Joint Laboratory for Human Microbiome, Institute of Advanced Technology, University of Science and Technology of China, Hefei, China; ^3^Anhui Provincial Key Laboratory of Digestive Disease, The First Affiliated Hospital of Anhui Medical University, Hefei, China

**Keywords:** fecal microbiota transplantation, long-term follow up, microbiota, inflammatory cytokines, ulcerative colitis

## Abstract

**Background:**

Ulcerative colitis (UC) is a multi-factor disease characterized by alternating remission periods and repeated occurrence. It has been shown that fecal microbiota transplantation (FMT) is an emerging and effective approach for UC treatment. Since most existing studies chose adults as donors for fecal microbiota, we conducted this study to determine the long-term efficacy and safety of the microbiota from young UC patient donors and illustrate its specific physiological effects.

**Methods:**

Thirty active UC patients were enrolled and FMT were administered with the first colonoscopy and two subsequent enema/transendoscopic enteral tubing (TET) practical regimens in The First Affiliated Hospital of Anhui Medical University in China. Disease activity and inflammatory biomarkers were assessed 6 weeks/over 1 year after treatment. The occurrence of adverse events was also recorded. The samples from blood and mucosa were collected to detect the changes of inflammatory biomarkers and cytokines. The composition of gut and oral microbiota were also sampled and sequenced to confirm the alteration of microbial composition.

**Results:**

Twenty-seven patients completed the treatment, among which 16 (59.3%) achieved efficacious clinical response and 11 (40.7%) clinical remission. Full Mayo score and calprotectin dropped significantly and remained stable over 1 year. FMT also significantly reduced the levels of C-reactive protein (CRP), interleukin-1 beta (IL-1β), and interleukin-6 (IL-6). The gut microbiota altered significantly with increased bacterial diversity and decreased metabolic diversity in responsive patients. The pro-inflammatory enterobacteria decreased after FMT and the abundance of Collinsella increased. Accordingly, the altered metabolic functions, including antigen synthesis, amino acids metabolism, short chain fatty acid production, and vitamin K synthesis of microbiota, were also corrected by FMT.

**Conclusion:**

Fecal microbiota transplantation seems to be safe and effective for active UC patients who are nonresponsive to mesalazine or prednisone in the long-term. FMT could efficiently downregulate pro-inflammatory cytokines to ameliorate the inflammation.

## Introduction

Ulcerative colitis (UC) is a chronic nonspecific intestinal inflammatory disease, mainly involving the colonic mucosa and submucosa with continuous distribution. Its clinical manifestations mainly include diarrhea, abdominal pain, mucous pus, and blood stool. Patients with UC may present different degrees of systemic symptoms and intestinal manifestations and have a risk of developing colorectal cancer through the inflammation-proliferation-cancer sequence pathway, which requires lifelong monitoring (Ananthakrishnan, 2015; Abdalla et al., 2017).

It is widely believed that the over-activation of the immune system and the dysbiosis of intestinal microbiota are associated with the intestinal inflammation in genetically susceptible people although the exact mechanism of pathogenesis of UC remains unclear (Chu et al., 2016; Hu et al., 2020). Gut microbiota is generally regarded as a covert metabolic and immune organ, participating in many physiological processes of host, including digestion and metabolism, adjustment of the epithelial barrier, development and regulation of host immune system, as well as the protection against pathogens (Pickard et al., 2017). A previous study has shown the link between the perturbation of intestinal microecological balance and the adverse bacterial community structure of UC patients, which is mainly manifested as the significant decrease of Bacteroidetes and Firmicutes, and the increase of Proteobacteria and Actinomycetes (Browne and Kelly, 2017). Although no specific pathogen has been identified as the sole cause of UC, studies have revealed the important role of the altered diversity of microbiota in intestinal inflammation (Ott et al., 2004; Chang, 2020). For instance, UC patients exhibited a decreased diversity of gut microbiota, including the reduction of Faecalibacterium prausnitzii, Clostridium clusters IV and XIVa, Bifidobacterium, Bacteroides, Roseburia, and Eubacterium rectale (Zhang et al., 2017). Therefore, it is potentially feasible to treat UC *via* improving the structure of gut microbiota communities in UC patients. Conversely, people suffering from inflammatory bowel disease (IBD) often showed oral symptoms like oral ulcer, dry mouth, and aphthous stomatitis (Jose and Heyman, 2008; Veloso, 2011), which indicate the potential correlation between oral microbiota and IBD manifestations. Although there have been some studies in this field (Said et al., 2014; Rautava et al., 2015; Xun et al., 2018), the information about the oral microbiota from IBD patients is still limited.

Fecal microbiota transplantation (FMT) is a procedure placing fecal microbiota from a healthy donor into a patient’s intestine to rectify microbiome imbalance (Gupta and Khanna, 2017), which has been shown to be highly successful in the treatment of Clostridium difficile infection (CDI) and has been approved by U.S. Food and Drug Administration (FDA) for clinical application of CDI (Surawicz et al., 2013). However, the efficacy and safety of FMT applied to UC treatment remain unclarified. To date, four high-quality randomized controlled studies (RCT) have reported the effectiveness of FMT in the treatment of active UC, among which three were confirmed to have significant improvement following FMT, with a clinical remission achievement of 24%–44% of patients when compared with the control group (5%–20%; Moayyedi et al., 2015; Paramsothy et al., 2017; Costello et al., 2019). A meta-analysis involving 168 FMT clinical studies showed that the final remission rate of FMT in UC could reach 39.6% and the overall incidence of adverse events was less than 1% (Lai et al., 2019). However, there is one RCT study that failed to illustrate the beneficial effect of FMT on UC (Rossen et al., 2015). Hence, the effectiveness of FMT for UC is still controversial and differences in the definition of clinical outcomes, donor selection, fecal microbiota preparation, and infusion delivery in these studies may be responsible for the inconsistent conclusions. In addition, the long-term influence of FMT on gut microbiota also needs to be monitored.

The purpose of this study is to explore the long-term efficacy and safety of FMT in the treatment of active UC using fecal microbiota from young donors. Combined with detection of host immune-related markers and long-term examination on microbiota, this study also contributes to a deeper understanding of the concrete mechanism of FMT in UC.

## Materials and Methods

### Patients

This is a single-center, open-label clinical study in the First Hospital of Anhui Medical University from April 2018 to March 2020. The inclusion criteria are as follows: (1) age 18–70; (2) biopsy confirmed UC with Mayo score of 4–10 and Mayo endoscopic score ≥ 1; and (3) received stable doses of mesalazine or prednisone for at least 3 months before enrollment. Exclusion criteria included: (1) pregnant or lactating, (2) concurrent with other infections, such as CDI or cytomegalovirus infection, (3) accompanied with other severe organic diseases, such as acute cerebrovascular disease, acute myocardial infarction, moderate to severe chronic obstructive pulmonary disease, and malignant tumor, (4) receiving any drugs that may affect the results of the study during the study period, including antibiotics, probiotics, and prebiotics, and (5) any patients considered inappropriate for inclusion by the researcher. The patient’s history and clinical characteristics were recorded at baseline.

### Ethics

The study was approved by the Hospital Ethics Committee of Anhui Medical University and registered in the department of Chinese Clinical Trial (registration No. ChiCTR1900022273). All patients provided written informed consent.

### Donor and Material Preparation

Forty-two donors were recruited from healthy, unrelated boys aged 8–14 years and screened for fecal and serological screening. The exclusion criteria for donors included: (1) antibiotic use within 3 months prior to donation, (2) a history of gastrointestinal disorders or familial gastrointestinal diseases, (3) autoimmune or other immune-mediated diseases, metabolic syndrome, malignancy, emaciation, or overweight. The serological pathogens including hepatitis A, B, C, and E, HIV, Rotavirus, Adenovirus, Treponema pallidum, and enteric pathogens including Salmonella, Campylobacter, Yersinia, Shigella, CDI, and parasites detected. The screens for vancomycin-resistant, extended spectrum beta-lactamase, and carbapenemase producing bacteria were also performed.

The preparation of fecal microbiota was performed in USTC-IAT and Chorain Health Joint Laboratory for Human Microbiome. Briefly, the sterile normal saline was added to homogenize the stool. The slurry was filtered using stainless steel sieves then centrifuged. The microbiota were resuspended with normal saline again and added with glycerol to a final concentration of 12.5% as freeze protectant. All procedures were performed under strict anaerobic conditions to protect the anaerobes. The final fecal microbiota was labeled and stored at −80°C before use.

### Transplantation Procedure

All patients who met the inclusion criteria underwent standard intestinal cleansing using polyethylene glycol before colonoscopy without the use of antibiotics. During the first colonoscopic FMT treatment, 120 ml fecal microbiota from three different donors was pooled then injected into the terminal ileum. For the next 4 days, patients received two subsequent FMT at the same dose. The infusion route depended on the Montreal classification of extent of UC: enema was used for E1 and E2 patients, and TET was used for E3 patients. After each infusion, the patient was encouraged to keep a lying position for 4–6 h to facilitate the successful colonization.

### Outcome Measurement

Within 24 h after FMT infusion, the patient’s vital signs (body temperature, blood pressure, breathing, and heart rate) were closely monitored. At week 1, 3, and 5 after FMT, patients received follow-up calls for adverse events (AE). New symptoms and the exacerbation of previous symptoms were recorded as AE. An AE that was disease spreading, fatal, life threatening, or required professional intervention requiring hospitalization or prolonged hospital stays, infertility congenital anomaly, permanent disability, or disfigurement was regarded as a serious AE. For short-term efficacy assessment, the full Mayo score and Mayo endoscopic score as well as AE were assessed at week 6. Mayo score ≤ 2 and the endoscopic Mayo score ≤ 1 were considered indicative of clinical remission. A reduction in the total Mayo score of ≥3 at week 6 was considered clinical response. The clinical biomarkers were also detected at this time point. For long-term efficacy assessment, the full Mayo score and Mayo endoscopic score as well as AE were assessed 1 year after FMT. The gut and oral microbiota were sampled and sequenced at both time points.

### Calprotectin Measurement

About 50–100 mg of patient stool samples were taken and a certain proportion of the extraction solution was added (w/v = 1:49) and mixed evenly. The 2 ml mixture was absorbed for centrifugation (3,000 *g* × 5 min), and then the supernatant was collected and processed according to the manufacturer’s instructions (Buhlmann Company, Switzerland).

### Biomarker Detection

The blood sample was centrifuged (3,000 *g* × 10 min) to obtain the supernatant, and the conditioned serum was collected and stored at −80°C until further use. The contents of interleukin-1 beta (IL-1β), interleukin-6 (IL-6), interleukin-10 (IL-10), and tumor necrosis factor alpha (TNF-α) in the sera were determined by the ELISA kits (NeoBioscience, China), and serum levels of vitamin D were measured by using DIA source 25OH Vitamin D total-RIA-CT kit (Louvain-La-Neuve, Belgium) according to the manufacturer’s instructions.

The colon mucosa of the most severe lesions was sampled under endoscopy. IL-1β, IL-6, IL-10, TNF-α, and Vitamin D Receptor (VDR) levels were measured using immunohistochemical analysis. The following primary antibodies were used: IL-1β (GB11113, Servicebio), IL-6 (21865-1-AP, Proteintech), IL-10 (20850-1-AP, Proteintech), TNF-α (60291-1-IG, Proteintech), and VDR (Ab3508, Abcam). Three areas were chosen randomly and then the mean optical density was measured using a light microscope (Nikon Eclipse ci, Japan) and Image-Pro Plus6.0 (Media Cybemetics, United States).

### 16S rRNA Gene Sequencing Sample Collection and Sequencing

All intestinal and oral samples were collected in the hospital. Briefly, fresh stool samples were collected using a sterile cotton swab and placed in a 2 ml sterile sampling tube. Fresh saliva samples were also collected and placed in a 2 ml sterile sampling tube. Samples were then sent to Novogene sequencing center in Tianjin, China and G-BIO sequencing center in Hangzhou, China for DNA extraction and sequencing. The genomic DNA was extracted using QIAamp Fast DNA Stool Mini Kit (Qiagen, Hilden, Germany), and the 16S rRNA gene PCR primers (341F: CCTAYGGGRBGCASCAG and 806R: GGACTACNNGGGTATCTAAT) were used to amplify 16S rRNA gene V3–V4 hypervariable region using total DNA from each sample as a PCR template. The library was constructed using TruSeq DNA PCR-Free Sample Preparation Kit, and then the samples were sequenced using the NovaSeq system (Illumina) with a 2 × 250-base pair protocol.

### Bioinformatics Analysis of 16S rRNA Gene Sequencing

First, the primer region of 16S rRNA gene sequencing raw data was removed using Cutadapt (version 1.18; Martin, 2011). The paired sequences were merged using Vsearch (version 2.14.1) with default parameters (Rognes et al., 2016). Then, the merged reads were analyzed using QIIME2 (version 2019.10; Bolyen et al., 2018). DADA2 was used to filter the low-quality merged reads and construct a feature table (100% identity; Callahan et al., 2016). The taxonomy was assigned using the Greengenes database (version 13.8; McDonald et al., 2012). Alpha and beta diversities were performed by QIIME2 and the MetaCyc metabolic pathway of bacteria was predicted using PICRUSt2 (version 2.3.0; Douglas et al., 2020). The code was uploaded to github.^1^ Differential abundances of taxa were compared using LEfSe (Segata et al., 2011).

### Statistical Analysis

Normally distributed data were analyzed using the paired *t* test and unpaired *t* test, and expressed as mean (SD). Non-normally distributed data were analyzed using Mann–Whitney U test and Wilcoxon rank sum test, and expressed as median (IQR). Values of *p* were corrected with BH method for multiple comparisons. Data analysis and visualization in this study were carried out using R version 4.0.0 software, utilizing tidyverse and agricolae packages.

## Results

### Clinical Outcomes

**Figure 1:**
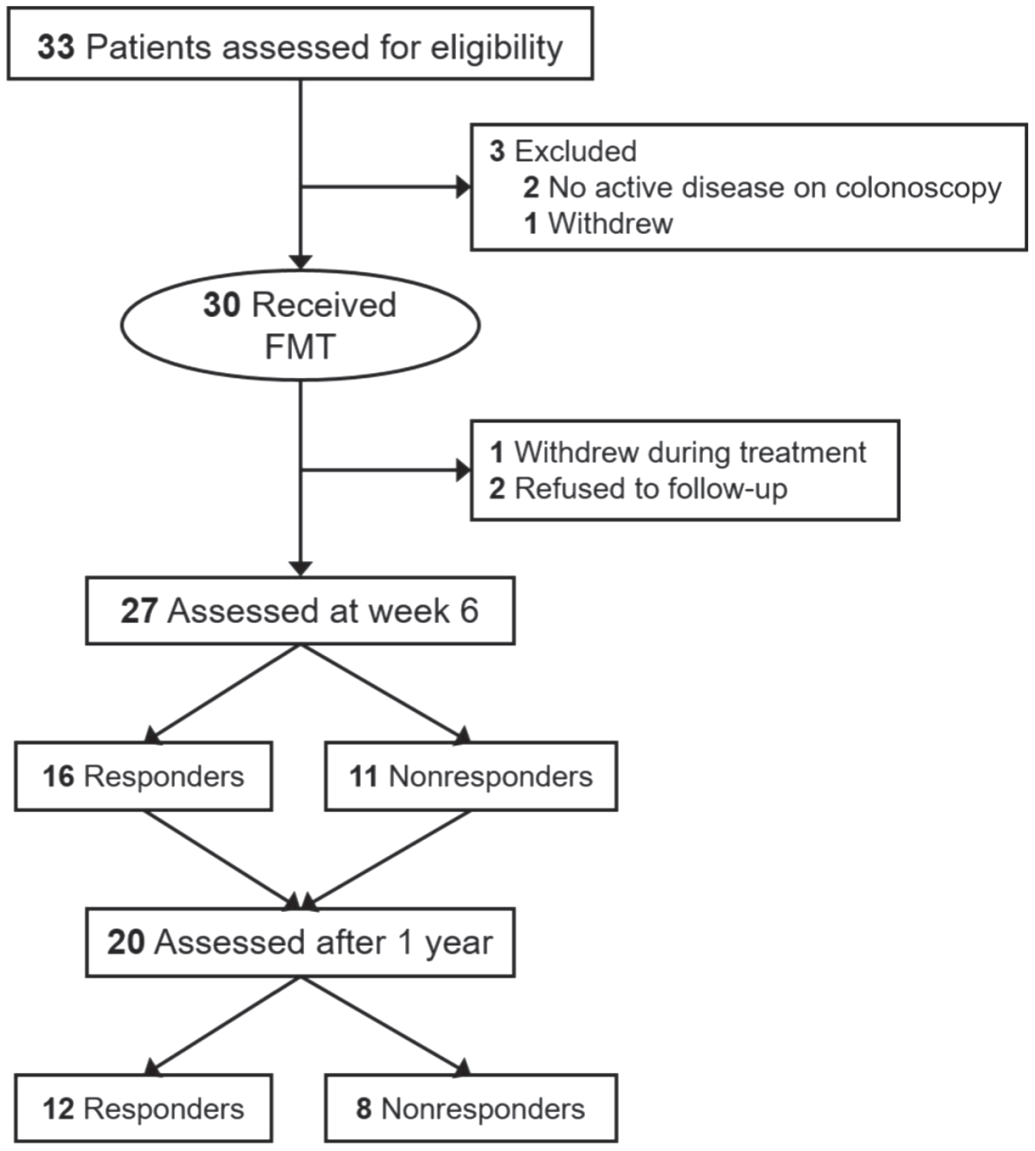
Flow of patients of fecal microbiota transplantation (FMT) for ulcerative colitis.

**Figure 2:**
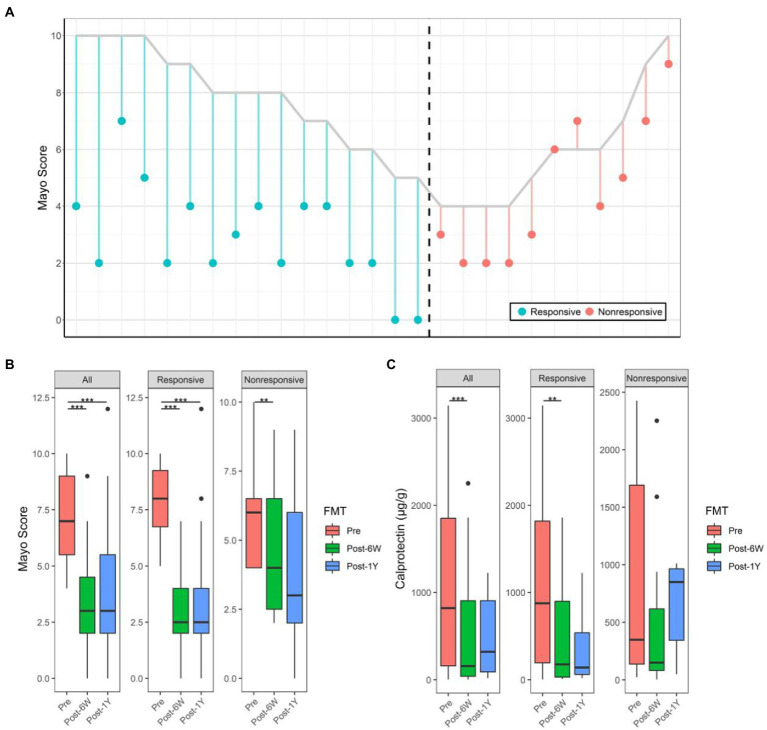
Alterations of full Mayo score and calprotectin. **(A)** Change of full Mayo score of all patients 6 weeks after FMT. The vertical parallel line plot shows changes in Mayo score for each individual patient. For each patient who received FMT, the line starts at his/her pre-FMT full Mayo score and ends at his/her FMT full Mayo score 6 weeks after FMT. The color shows the response of each patient. **(B)** The Mayo score of patients before, 6 weeks after, and 1 year after FMT. **(C)** The calprotectin levels of patients before, 6 weeks after, and 1 year after FMT. Boxplots present the median and interquartile range (25–75th percentiles) for each group with whisker length equal to 1.5 interquartile range. The colors show the group of patients. Statistically significances are also indicated: ^**^p < 0.01, ^***^p < 0.001.

Twenty-seven patients successfully completed the entire trial (Figure 1) and their baseline clinical characteristics are presented in Table 1. Sixteen patients (59.3%) achieved a clinical response (full Mayo score decreased ≥3) and 11 patients (40.7%) were in clinical remission by week 6 (full Mayo score ≤ 2 and mayo endoscopy subscore ≤ 1). The short-term change in full Mayo score for each participant is represented in Figure 2A. The mean full Mayo score dropped by 4.94 (95% CI 4.17–5.70) on average in the responsive group and by 1.36 (95% CI 0.67–2.05) in the nonresponsive group at week 6 after FMT. In total, the mean full Mayo score of all 27 patients dropped by 3.48 (95% CI 2.61–4.35; Figure 2B). The long-term efficacy was also measured 1 year after FMT. The results showed the decrease of full Mayo scores could remain stable for more than 1 year in both responsive and nonresponsive patients. By comparison, the levels of calprotectin at all three time points (before FMT, 6 weeks after FMT, and 1 year after FMT), showed that FMT could effectively downregulate the level of calprotectin (Figure 2C), although the decrease is not statistically significant (*p* = 0.0674) in nonresponsive patients. A two-way ANOVA was also performed to analyze the effect of FMT and response on these two clinical outcomes. The results revealed that there was a statistically significant interaction between the effects of FMT but not the response (Table 2).

**Table 1:** Baseline characteristics of recipients.

	Pre-FMT	Responders	Nonresponders	*p* value
Age (Mean, SD)	47.48 ± 12.34	48.44 ± 12.08	46.09 ± 13.16	0.642
Sex, *N* (%)				
Women	10 (37%)	8 (29.63%)	2 (7.41%)	0.124
Men	17 (63%)	8 (29.63%)	9 (33.33%)	
Extent of disease, *N* (%)				
Proctitis	2 (7)	0	2	0.058
Left sided colitis	21 (78)	12	9	
Pancolitis	4 (15)	4	0	

**Table 2:** Corrected *p* values of two-way ANOVA analysis.

Name	FMT	Response	FMT*Response
**Clinical indices**			
Full Mayo score	3.05E-06	0.959496179	0.068477498
Calprotectin	0.016552863	0.959496179	0.48252139
CRP	0.347700137	0.442741139	0.764509514
ESR	0.730812035	0.172951201	0.919061664
IL-1_Histo	0.023727928	0.442741139	0.919061664
IL-6_Histo	0.000965475	0.806509332	0.919061664
IL-10_Histo	0.730812035	0.951145899	0.213197621
TNF-alpha_Histo	0.529979285	0.442741139	0.389165106
VitD_Histo	0.015244513	0.831544693	0.691447528
IL-1_Serum	0.385058137	0.544553385	0.882812366
IL-6_Serum	0.347700137	0.442741139	0.691447528
IL-10_Serum	0.877379654	0.597396304	0.919061664
TNF-alpha_Serum	0.730812035	0.442741139	0.882812366
VitD_Serum	0.877379654	0.806509332	0.882812366
**Gut microbiota diversity**			
Observed_OTUs	2.05E-07	0.955249274	0.93134377
Evenness	0.035567919	0.955249274	0.9015684
Shannon	6.57E-05	0.955249274	0.9015684
path_Observed_OTUs	0.000613481	0.955249274	0.9015684
path_Evenness	0.055638589	0.955249274	0.93134377
path_Shannon	0.002270375	0.955249274	0.93134377
**Oral microbiota diversity**			
Observed_OTUs-Oral	0.001176824	0.955249274	0.9015684
Evenness-Oral	0.115613198	0.955249274	0.9015684
Shannon-Oral	0.394387053	0.955249274	0.9015684
path_Observed_OTUs-Oral	6.57E-05	0.955249274	0.9015684
path_Evenness-Oral	0.333186925	0.955249274	0.786279492
path_Shannon-Oral	2.94E-05	0.955249274	0.93134377

Although no serious AE were observed, four patients had minor adverse reactions. The first patient developed blackened tongue coating within 1 day after treatment, while the second patient presented transient fever and symmetrical erythema on both lower limbs and posterior back, 4 h and 1 week after transplantation, respectively. The forementioned signs of both patients subsided after conservative care during the follow-up period. The third patient showed perianal abscess after transplantation, which improved after antibiotic treatment. The last patient presented with an exacerbation of colitis during the follow-up period and was hospitalized for anti-inflammatory and hormone therapy. In addition, no correlation between the outcomes (both clinical response and adverse events) and individual donor was found.

### Influence of FMT on Gut Microbiota in Patients

**Figure 3:**
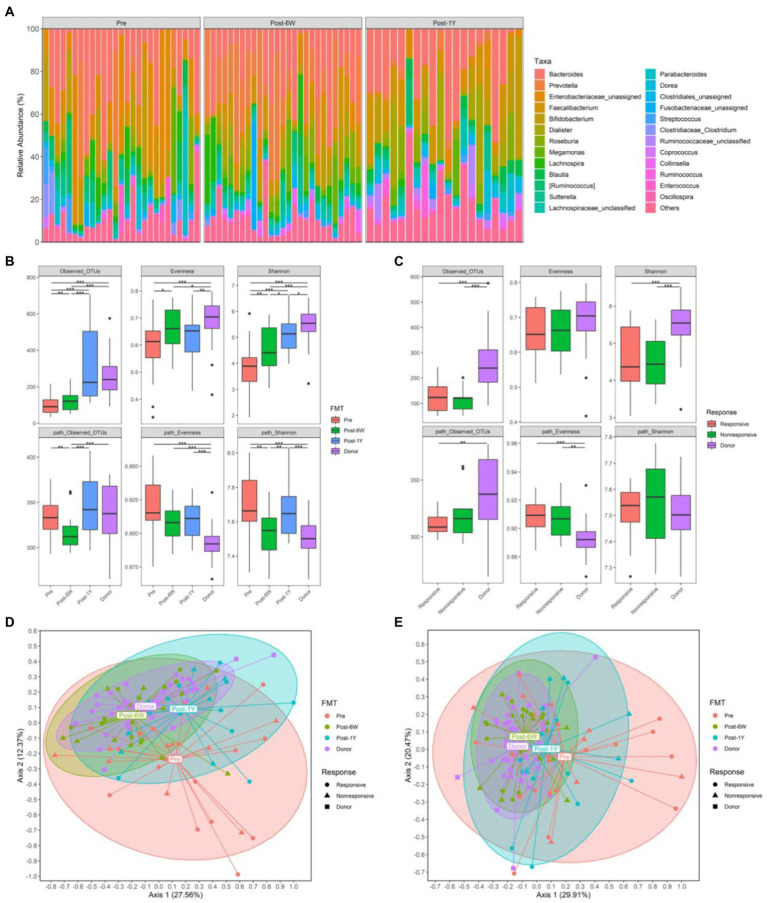
The gut microbiota from all patients. **(A)** The relative abundance of gut microbiota in each patient throughout the clinical study. **(B)** The alpha diversities of all patients and donors throughout the clinical study. **(C)** The alpha diversities of responsive patients, non-responsive patients, and donors 6 weeks after FMT. **(D)** PCoA based on weighted UniFrac matrix of bacterial taxonomy. **(E)** PCoA based on Bray-Curtis matrix of predicted pathways. Boxplots present the median and interquartile range (25–75th percentiles) for each group with whisker length equal to 1.5 interquartile range. Statistically significances are also indicated: ^*^p < 0.05, ^**^p < 0.01, and ^***^p < 0.001.

The microbiota compositions of each patient throughout the clinical study were analyzed and presented at genus level (Figure 3A). The taxonomic and metabolic alpha diversities, including observed OTUs, Pielou’s evenness index, and Shannon diversity index between different groups, were calculated based on the OTU table and representative sequences generated by DADA2 (the statistics of this process were listed in Table 3). The taxonomic observed OTUs and Shannon index in patients were lower than that in the donor (Figure 3B). FMT continuously enhanced these two alpha diversities after treatment. Surprisingly, the metabolic alpha diversities in patients were significantly higher than that in the donor in spite of low bacterial diversities. FMT also resulted in changes towards normal levels, although there was a rebound in the trend after 1 year. Patients were divided into two groups based on their clinical response as mentioned above. Unexpectedly, no significant difference of the six alpha diversities was noted between the responsive group and the nonresponsive group at week 6 (Figure 3C), which implies the alpha diversities might not be implicated in clinical response. The two-way ANOVA results also verified the influence of FMT instead of response (Table 2).

**Table 3:** Statistics of DADA2 process.

Sample-id	Input	Filtered	Percentage of input passed filter	Denoised	Non-chimeric	Percentage of input non-chimeric
AX105	29,983	29,357	97.91	29,072	28,774	95.97
AX108	27,296	26,563	97.31	26,401	25,066	91.83
AX183	25,841	25,151	97.33	24,415	21,908	84.78
AX185	24,474	23,578	96.34	22,877	22,836	93.31
AX186	25,803	25,146	97.45	25,059	25,042	97.05
AX187	23,654	22,945	97	22,493	22,151	93.65
AX188	23,135	22,235	96.11	22,213	22,148	95.73
AX189	26,040	25,165	96.64	24,782	24,212	92.98
AX190	26,514	25,705	96.95	25,396	24,572	92.68
AX191	24,381	23,595	96.78	23,389	23,040	94.5
AX192	23,889	23,256	97.35	23,175	23,151	96.91
AX193	22,621	21,795	96.35	21,485	21,272	94.04
AX194	24,836	24,271	97.73	23,924	19,870	80
AX195	25,187	24,477	97.18	24,140	20,219	80.28
AX196	21,774	21,023	96.55	20,775	20,561	94.43
AX197	27,039	26,324	97.36	26,123	23,449	86.72
AX198	22,583	21,835	96.69	21,783	21,783	96.46
AX199	25,533	24,722	96.82	24,568	22,305	87.36
AX200	26,361	25,525	96.83	25,264	24,629	93.43
AX201	23,644	22,830	96.56	22,422	21,455	90.74
AX202	28,142	27,378	97.29	27,226	27,133	96.41
AX203	26,313	25,462	96.77	25,400	25,357	96.37
AX204	22,017	21,453	97.44	21,092	20,404	92.67
AX205	25,658	25,033	97.56	24,682	23,865	93.01
AX218	28,538	27,908	97.79	27,492	27,038	94.74
AX219	29,233	28,585	97.78	28,042	25,481	87.17
AX220	31,621	31,002	98.04	30,729	30,542	96.59
AX221	27,740	27,171	97.95	26,820	26,578	95.81
AX222	28,922	28,285	97.8	27,932	27,060	93.56
AX223	29,356	28,742	97.91	28,275	27,776	94.62
AX237	29,285	28,436	97.1	27,828	27,006	92.22
AX89	29,078	28,364	97.54	28,178	27,452	94.41
AX90	30,345	29,594	97.53	29,109	28,728	94.67
AX91	28,737	27,995	97.42	27,543	26,349	91.69
AX92	31,272	30,550	97.69	30,204	29,688	94.93
AX93	31,914	31,121	97.52	30,663	30,418	95.31
AX94	26,137	25,498	97.56	24,537	23,888	91.4
AX95	30,791	30,026	97.52	29,634	29,226	94.92
AX96	29,774	29,067	97.63	28,984	28,777	96.65
CCL.C	39,548	32,953	83.32	28,879	26,034	65.83
CCL.K	40,007	31,407	78.5	28,456	26,491	66.22
CN.C	74,827	66,512	88.89	59,483	51,271	68.52
CN.K	60,205	51,137	84.94	47,316	40,943	68.01
FLS.C	70,579	62,242	88.19	55,838	51,483	72.94
FLS.K	35,754	27,417	76.68	23,745	21,038	58.84
GQJ.C	35,747	28,535	79.82	24,497	22,441	62.78
GQJ.K	48,471	38,075	78.55	35,665	33,495	69.1
GTAA917	94,017	91,203	97.01	84,242	68,863	73.25
GTAB917	76,086	73,749	96.93	67,693	48,301	63.48
GTAC920	91,839	88,836	96.73	81,492	52,122	56.75
GTAD922	121,808	118,231	97.06	112,856	93,935	77.12
GTAE1009	98,278	95,132	96.8	86,948	67,386	68.57
GTAF923	65,734	63,684	96.88	57,577	45,317	68.94
GTAG1009	84,974	82,398	96.97	75,266	60,098	70.73
GTN1211	69,202	65,344	94.43	58,770	50,567	73.07
GTR124	58,951	56,458	95.77	50,093	42,219	71.62
GTS1210	72,719	69,587	95.69	64,192	57,459	79.02
GTW927	80,856	78,438	97.01	69,565	45,248	55.96
GTX908	94,363	91,296	96.75	84,195	65,260	69.16
GZC.C	60,985	51,653	84.7	46,107	37,539	61.55
GZC.K	58,292	47,595	81.65	42,192	38,458	65.97
HGT	22,684	22,060	97.25	20,060	17,209	75.86
HZY	28,082	27,315	97.27	26,521	25,056	89.22
JXY.C	74,113	67,473	91.04	62,849	53,135	71.69
JXY.K	61,784	53,642	86.82	50,508	44,709	72.36
KR	26,567	25,835	97.24	25,038	24,679	92.89
LCH.C	37,971	31,415	82.73	26,000	23,848	62.81
LCH.K	40,090	30,930	77.15	28,231	26,749	66.72
LSH	30,211	29,441	97.45	28,898	28,236	93.46
LSQ.C	37,176	30,653	82.45	26,181	23,269	62.59
LSQ.K	58,289	47,923	82.22	44,611	36,848	63.22
LXH.C	66,978	60,755	90.71	56,120	44,859	66.98
LXH.K	52,420	43,021	82.07	41,399	40,487	77.24
LZX.C	75,601	70,615	93.4	65,276	53,412	70.65
LZX.K	42,777	33,608	78.57	30,532	28,851	67.45
NAYF0001	65,708	55,236	84.06	54,084	53,558	81.51
NAYF0002	69,966	61,473	87.86	59,255	57,856	82.69
NAYF0003	71,314	62,848	88.13	61,386	60,159	84.36
NAYF0004	64,769	56,754	87.63	54,827	52,443	80.97
NAYF0005	59,420	51,893	87.33	50,411	49,623	83.51
NAYF0006	70,403	61,747	87.71	57,016	50,270	71.4
NAYF0007	71,823	62,746	87.36	59,658	48,224	67.14
NAYF0008	69,922	61,305	87.68	59,113	56,684	81.07
NAYF0009	72,925	64,153	87.97	62,778	61,702	84.61
NAYF0010	63,548	55,523	87.37	54,158	50,168	78.95
NAYF0011	57,529	49,318	85.73	47,854	43,532	75.67
NAYF0012	72,206	63,935	88.55	59,807	53,781	74.48
NAYF0013	68,853	60,686	88.14	59,405	58,490	84.95
NAYF0014	64,526	57,369	88.91	55,850	54,401	84.31
NAYF0015	67,537	59,388	87.93	58,056	54,023	79.99
NAYF0016	66,588	59,071	88.71	57,325	55,535	83.4
NAYF0017	68,074	60,146	88.35	58,209	57,126	83.92
NAYF0018	61,711	54,166	87.77	52,977	52,479	85.04
NAYF0019	62,616	55,523	88.67	54,498	54,063	86.34
NAYF0020	73,741	65,952	89.44	64,419	63,421	86.01
NAYF0021	62,954	56,668	90.01	54,649	53,536	85.04
NAYF0022	71,903	64,551	89.78	62,471	61,743	85.87
NAYF0023	74,305	66,411	89.38	65,094	60,162	80.97
NAYF0024	62,278	55,212	88.65	53,616	52,771	84.73
NAYF0025	71,301	64,255	90.12	62,946	61,135	85.74
NAYF0026	73,386	65,723	89.56	63,755	62,764	85.53
NAYF0027	75,736	67,955	89.73	65,787	64,069	84.6
NAYF0028	64,630	57,217	88.53	55,879	55,095	85.25
NAYK0001	56,072	52,683	93.96	49,144	46,930	83.7
NAYK0005	53,262	49,853	93.6	46,851	44,043	82.69
NAYK0006	53,844	50,630	94.03	47,515	43,439	80.68
NAYK0007	70,301	65,649	93.38	62,123	57,644	82
NAYK0009	69,989	65,448	93.51	62,065	59,026	84.34
NAYK0010	55,159	51,824	93.95	48,539	44,297	80.31
NAYK0011	68,798	63,898	92.88	59,264	54,040	78.55
NAYK0012	75,927	68,909	90.76	64,882	58,449	76.98
NAYK0013	70,485	65,138	92.41	60,680	51,357	72.86
NAYK0014	62,012	57,488	92.7	52,970	48,067	77.51
NAYK0015	68,025	62,928	92.51	59,007	51,998	76.44
NAYK0016	74,992	69,847	93.14	64,449	54,073	72.11
NAYK0017	75,771	70,542	93.1	66,327	58,269	76.9
NAYK0019	69,989	64,916	92.75	60,863	56,005	80.02
NAYK0020	77,982	71,844	92.13	67,242	58,205	74.64
NAYK0022	85,618	79,566	92.93	75,469	62,046	72.47
NAYK0023	76,164	70,758	92.9	66,787	49,602	65.13
NXG.C	43,572	35,513	81.5	31,858	29,764	68.31
NXG.K	44,610	36,539	81.91	32,963	31,181	69.9
SXF.C	42,399	34,945	82.42	30,344	27,109	63.94
SXF.K	45,877	36,971	80.59	33,390	32,186	70.16
WCQ	30,858	30,019	97.28	29,353	28,623	92.76
WHT	31,431	30,515	97.09	29,903	27,109	86.25
XA	29,954	29,125	97.23	28,148	27,858	93
XDC.C	75,033	71,326	95.06	64,782	53,875	71.8
XDC.K	61,580	51,943	84.35	49,571	47,137	76.55
XHY	24,804	24,128	97.27	22,369	20,707	83.48
XSY.C	60,943	52,388	85.96	47,656	40,231	66.01
XSY.K	42,293	33,967	80.31	29,300	28,405	67.16
YCY.C	36,506	29,285	80.22	25,885	21,377	58.56
YCY.K	35,486	27,698	78.05	23,428	23,139	65.21
YHY	25,858	25,127	97.17	24,640	23,985	92.76
YY.C	40,246	32,709	81.27	28,260	23,635	58.73
YY.K	41,994	34,119	81.25	29,882	28,127	66.98
YZH.C	36,442	29,278	80.34	25,438	20,613	56.56
YZH.K	43,675	35,483	81.24	30,698	28,868	66.1
YZL.C	41,205	33,519	81.35	29,063	25,729	62.44
YZL.K	68,400	60,040	87.78	55,110	48,276	70.58
ZPX.C	39,239	33,563	85.53	30,153	23,422	59.69
ZPX.K	55,131	46,142	83.7	42,825	37,347	67.74
ZXM.C	76,603	74,145	96.79	68,243	56,953	74.35
ZXM.K	34,510	26,737	77.48	23,019	22,332	64.71
ZY209992	70,719	65,173	92.16	58,129	50,136	70.89
ZY209993	56,124	51,565	91.88	45,891	36,517	65.06
ZY209994	56,119	51,527	91.82	47,296	36,868	65.7
ZY209995	65,293	60,231	92.25	54,731	42,541	65.15
ZY209996	57,975	53,644	92.53	49,097	32,100	55.37
ZY209997	62,687	58,090	92.67	54,758	38,886	62.03
ZY209998	85,212	79,264	93.02	75,841	74,643	87.6
ZY209999	55,297	51,140	92.48	46,749	37,553	67.91
ZYJK-1	29,587	28,962	97.89	28,652	28,197	95.3
ZYJK-2	28,961	28,380	97.99	27,900	27,225	94.01
ZYJK-3	28,985	28,374	97.89	27,458	26,891	92.78
ZYJK-5	26,788	26,312	98.22	26,241	25,677	95.85
ZYJK-6	31,074	30,572	98.38	29,848	28,779	92.61

Next, we performed principal coordinate analysis based on weighted UniFrac distance of taxonomy and Bray-Curtis distance of pathway. The results showed the taxonomic compositions in patients were distinct from that in donors before treatment (Figures 3D,E). FMT altered the taxonomic structures of patients at week 6 and the alteration could last for more than 1 year. When taking clinical response into account, the taxonomic structures of responsive and nonresponsive patients were the same throughout the entire clinical study. But the significant alteration was observed only in responsive patients. As for metabolic compositions, the changes were the same with those of taxonomic compositions.

### Alterations of Biomarkers and Their Correlation With Microbiota

**Figure 4:**
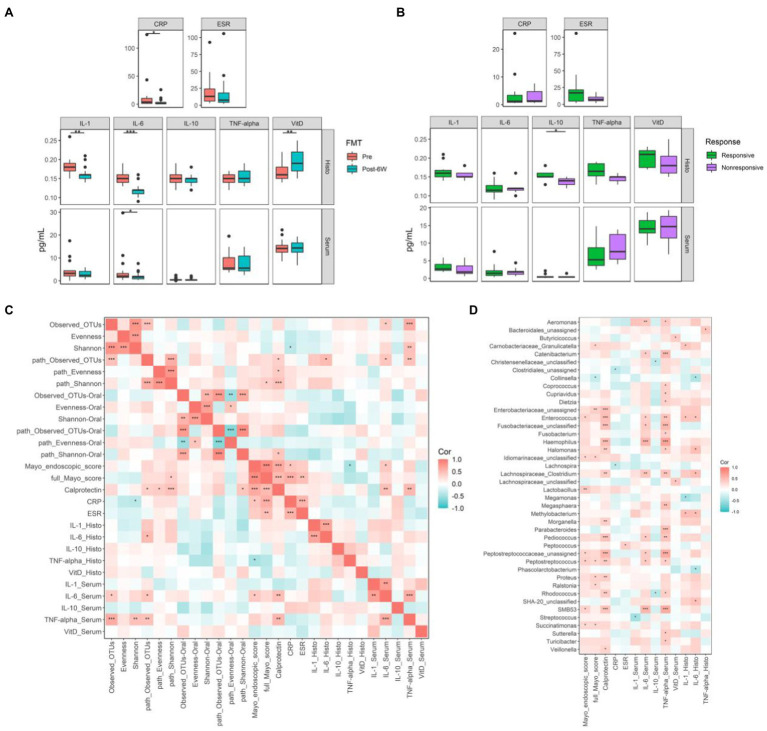
The biomarkers related to ulcerative colitis (UC) and their correlations with gut microbiota. **(A)** The biomarkers levels of all patients before and 6 weeks after FMT. **(B)** The biomarkers levels of responsive and nonresponsive patients 6 weeks after FMT. **(C)** The correlation coefficients between biomarkers and microbial diversities. **(D)** The correlation coefficients between biomarkers and gut microbiota at genus level. Boxplots present the median and interquartile range (25–75th percentiles) for each group with whisker length equal to 1.5 interquartile range. The colors show the group of patients. Statistically significances are also indicated: ^*^p < 0.05, ^**^p < 0.01, and ^***^p < 0.001. The Spearman correlation coefficients were calculated and labeled in the figure. Statistically significances are also indicated: ^*^p < 0.05, ^**^p < 0.01, and ^***^p < 0.001.

To delineate the concrete mechanism of FMT therapy, we measured several biomarkers closely related to UC, including C-reactive protein (CRP), erythrocyte sedimentation rate (ESR), IL-1β, IL-6, IL-10, TNF-α, and vitamin D at week 6 (Figures 4A,B). The reduction of CRP combined with the reduction of calprotectin mentioned above suggested the amelioration of inflammation and the improvement of tissue damage. As for cytokines, the expression of pro-inflammatory IL-1β and IL-6 in intestinal mucosa was significantly dampened after FMT, while only the level of IL-6 decreased significantly in serum. The expression of VDR in intestinal mucosa was found to be significantly elevated in response to FMT. Further comparison between responsive and nonresponsive groups showed the upregulation of IL-10 only in intestinal mucosa of responsive patients, which is the only difference between responsive and nonresponsive patients after treatment. Simple main effects analysis showed that FMT did have a statistically significant effect on the levels of IL-1β, IL-6, and VDR in intestinal mucosa (Table 2).

Subsequently, the Spearman correlation coefficients between these biomarkers and the alpha diversities of gut microbiota and specific genus were calculated (Figures 4C,D). The taxonomic observed OTUs positively correlated with IL-6 and TNF-α in serum. High Shannon index correlated with low CRP and high TNF-α, which is somehow contradictory. Conversely, high metabolic diversities seemed to correlate with more severe inflammation, although many coefficients are not statistically significant. Most identified genus-associated biomarkers indicated the exacerbation of disease, especially increased calprotectin and TNF-α in serum. In contrast, Collinsella was the only genus linked with the improvement of full Mayo score.

### Alterations of Taxonomic and Metabolic Compositions Following FMT

**Figure 5:**
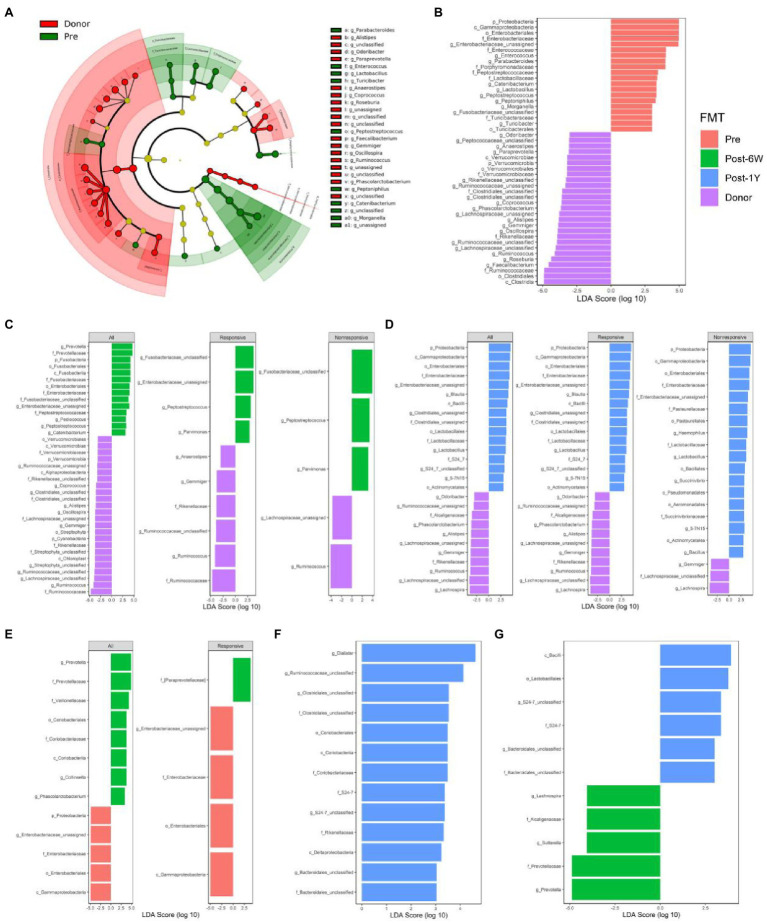
Comparison of differential taxonomic features at different time points. **(A)** The cladograms of differential taxa between UC patients and donors before FMT. Histograms of the LDA scores for taxonomic features differentially abundant between patients before FMT and donor **(B)**, patients 6 weeks after FMT and donor **(C)**, patients 1 year after FMT and donor **(D)**, patients before FMT and 6 weeks after FMT **(E)**, patients before FMT and 1 year after FMT **(F)**, and patients 6 weeks after FMT and 1 year after FMT **(G)**.

LEfSe analysis was performed to identify the differential taxa pathway as a result of FMT (cutoff value: LDA score ≥ 2, adjusted value of *p* < 0.05). We first compared the patients and donors to identify the key taxa involved in UC (Figures 5A,B). The results showed the enrichment of Proteobacteria, Enterococcus, and Turicibacter, and also the lack of Anaerostipes, Coprococcus, Roseburia, Faecalibacterium, Ruminococcus, and Gemmiger. FMT could increase the abundance of Prevotella, Collinsella, and Phascolarctobacterium, and reduce the abundance of pro-inflammatory bacteria, such as phylum Proteobacteria, class Gammaproteobacteria, and family Enterobacteriaceae (Figure 5E). It is not surprising that only the responsive group showed a decrease of Gammaproteobacteria and other taxa. The failure of statistical significance in the nonresponsive group might result from small difference and high variance of abundance. Patients after FMT still have many differential taxa compared with donors at week 6 (Figure 5C). The enriched taxa in donor group might reflect their low colonization efficiency, like Ruminococcus which was lower in both responsive and nonresponsive patients. The comparison between patients and donors after 1 year indicated the abundance of Proteobacteria and other pro-inflammatory taxa had a tendency to rise again (Figures 5D,F,G). Also, there were no differential taxa between the responsive and nonresponsive group after multiple comparison corrections throughout the study. The two-way ANOVA results did not identify the significant effect of response on any single genera, while three genera (S24-7_unclassified, Veillonella, and Enterobacteriaceae_unclassified) were proved to be influenced by FMT (*p* = 0.0030, 0.0254, 0.0254).

**Figure 6:**
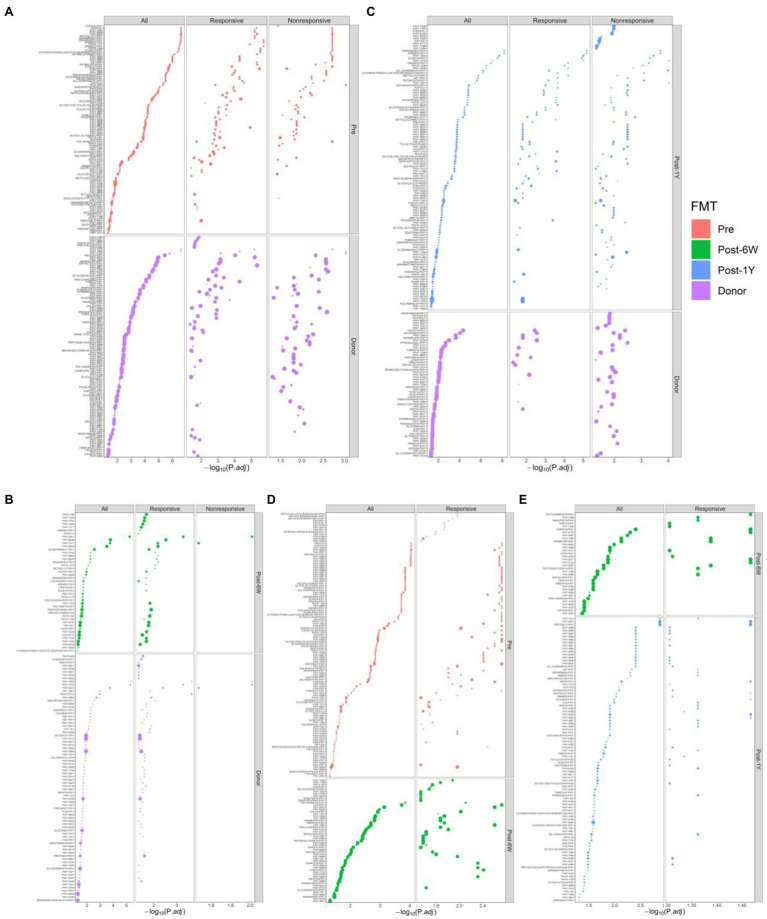
Enriched metabolic pathways modulated in response to FMT. Comparison of differential metabolic features at different time points: between patients before FMT and donor **(A)**, patients 6 weeks after FMT and donor **(B)**, patients 1 year after FMT and donor **(C)**, patients before FMT and 6 weeks after FMT **(D)**, and patients 6 weeks after FMT and 1 year after FMT **(E)**. The size of each point indicates the average relative abundance of this metabolic pathway.

PICRUSt2 were used to predict the metabolic pathways possessed by the gut microbiota based on 16S rRNA gene sequences. First, we compared the metabolic pathways in patients before FMT and donors (Figure 6A). The enriched pathways in patients included common antigen synthesis (ECASYN-PWY, ENTBACSYN-PWY, and LPSSYN-PWY), amino acids degradation (ARGDEG-PWY, AST-PWY, ORRGDEG-PWY, ORNDEG-PWY, and THREOCAT-PWY), and vitamin K synthesis (PWY-5838, PWY-5840, PWY-5850, etc.). The pathways enriched in donors included amino acids synthesis (HISTSYN-PWY, PWY-2942, PWY-5103, PWY-5104, COMPLETE-ARO-PWY, PWY-3001, PWY-5097, PWY-5101, THRESYN-PWY, and PWY-5505) short-chain fatty acids production (PWY-5676, PWY-5677, PWY-5100, and P163-PWY). Fatty acid biosynthesis (FASYN-INITIAL-PWY and FASYN-INITIAL-PWY) was only lower in the responsive group, indicating the decreased fatty acid might contribute to clinical response. Meanwhile, liposaccharides synthesis and heme production were only higher in the nonresponsive group which means these two metabolites might suppress remission. At week 6, common antigen synthesis decreased in response to FMT but was still higher than that in donor (Figures 6B,D). At the same time, higher amino acids degradation and vitamin K synthesis were corrected by FMT in all patients. There was no significant change observed in the nonresponsive group, while the short-chain fatty acid (P163-PWY, PWY-5676) and nucleotides synthesis (DENOVOPURINE2-PWY, PWY-7196, PWY-7199, PWY-7200, and PWY0-162) were enriched in the responsive group alone. There was no differential pathway between patients before and 1 year after FMT, which is understandable because PCoA results showed the difference of metabolic compositions between these two groups is barely significant (*p* = 0.0423). When compared with patients at week 6, responsive patients after 1 year showed reduction of amino acids synthesis (HISTSYN-PWY, PWY-2941, PWY-5088, PWY-5097, PWY-5104, SER-GLYSYN-PWY, and TRPSYN-PWY) and nucleotides synthesis (PWY-6609, PWY-7208, and PWY-7219; Figure 6E). Moreover, amino acid degradation and vitamin K synthesis in all patients rose again. When compared with donor, nonresponsive patients after 1 year exhibited some distinct pathways whose functions were mainly nucleotides synthesis (PWY-7228, PWY-6125, PWY-7196, PWY-7200, PWY-7228, and PWY-841). In addition, nonresponsive patients after 1 year also showed decreased production of some amino acids (ARGSYNBSUB-PWY, PWY-2942, and PWY-2942; Figure 6C). No pathway was associated with response, but 85.65% of pathways (382/446) were proved to be significantly associated with time.

### Analysis of Microbiota in Oral Cavity of UC Patients

**Figure 7:**
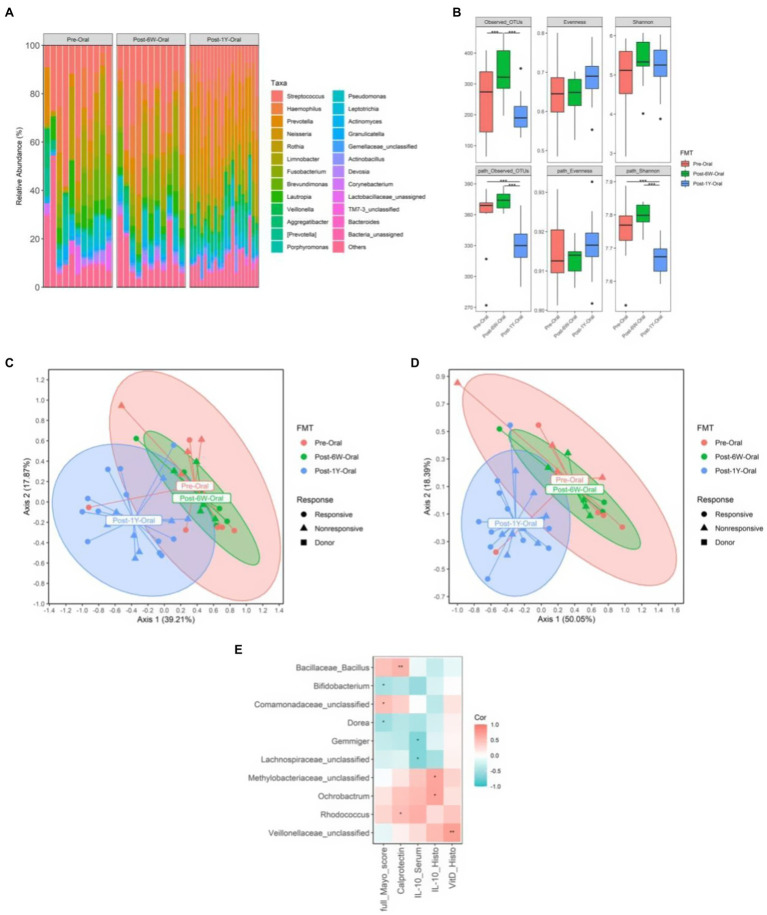
The change of oral microbiota from all patients. **(A)** The relative abundance of oral microbiota in each patient throughout the clinical study. **(B)** The alpha diversities of oral microbiota throughout the clinical study. **(C)** PCoA based on weighted UniFrac matrix of bacterial taxonomy. **(D)** PCoA based on Bray-Curtis matrix of predicted pathways. **(E)** The correlation coefficients between biomarkers and oral microbiota at genus level. Boxplots present the median and interquartile range (25–75th percentiles) for each group with whisker length equal to 1.5 interquartile range. The Spearman correlation coefficients were calculated and labeled in the figure. Statistically significances are also indicated: ^*^p < 0.05, ^**^p < 0.01, and ^***^p < 0.001.

To investigate the effect of FMT on ectopic microbiota, the microbes in the oral cavities of a portion of patients were collected. The oral microbiota composition of each patient was presented at genus level in Figure 7A. Analysis of diversities showed an increased number of observed OTUs after FMT but it dropped to the initial level after 1 year (Figure 7B). The increased metabolic alpha diversities also reduced after 1 year, suggesting FMT could only produce a short-term effect on patient’s oral microbiota instead of long-term effect on gut microbiota mentioned above. The beta diversities of oral microbiota based on taxonomic weighted UniFrac distance and metabolic Bray-Curtis distance were similar with the results of gut microbiota (Figures 7C,D) and only the responsive group exhibited significant alteration after FMT. But in concert with alpha diversities, this alteration was subverted after 1 year. There was no significant difference between responsive and nonresponsive group throughout the study. The Spearman correlation coefficients between clinical biomarkers and specific oral genus were also calculated (Figure 7E). Bifidobacterium, Dorea, Ochrobactrum, and some unclassified genus correlated with amelioration of inflammation (lower full Mayo score, higher IL-10, and VDR expression), while Bacillus, Gemmiger, and Rhodococcus correlated with exacerbation (higher calprotectin and lower IL-10). Among these genera, the two-way ANOVA results only proved Bacillus and Bifidobacterium were influenced by FMT (*p* = 0.0005 and *p* = 0.0066).

## Discussion

In this study, fecal microbiota from healthy young donors was proved to be an effective and safe strategy for the treatment of active ulcerative colitis. In general, an obvious clinical response was achieved in 16 patients (59.3%) and 11 patients (40.3%) were clinically relieved 6 weeks after FMT. Calprotectin is employed as a well-studied (systemic and fecal) inflammatory biomarker because of its stability, assay reproducibility, and low cost to guide diagnostic in IBD (Jukic et al., 2021). Full Mayo score and calprotectin both improved after FMT and maintained for more than 1 year, suggesting a reduction of inflammation and an amelioration of tissue injury. Several FMT studies conducted long-term follow-up of UC patients and showed 21.1% (23/109) and 25.7% (28/109) of response rates were observed after single and multiple FMTs at 6 months (Ding et al., 2019). One study showed 32% (12/38) achieved the primary outcomes and 42% (5/12) remained in remission at 12 months (Costello et al., 2019), while another study showed 87.1% (27/31) of patients receiving FMT and 66.7% (20/30) of patients receiving placebo every 8 weeks maintained clinical remission at week 48 (Sood et al., 2019). Patients well tolerated the operation and no serious adverse events were noted.

The gut microbiota of ulcerative colitis patients often showed low alpha diversity (Nusbaum et al., 2018; Ren et al., 2021), which was also proven in our study. The improvement of bacterial diversity and significant PCoA results indicated the successful correction of gut microbiota dysbiosis. Meanwhile, this study also illustrated high metabolic diversities in UC patients. Surprisingly, no difference was seen in either bacterial or metabolic diversities between responsive and nonresponsive groups, suggesting that the alpha diversities might not be the key factor for evaluation of the treatment effect. The significant alteration of taxonomy and metabolic pathways only occurred in the responsive group, which highlights the necessity of completely altered taxonomic and metabolic compositions for a clinical response.

Interleukin-1 beta and IL-6 have previously been reported to be upregulated in active UC specimens (Stevens et al., 1992; Mudter and Neurath, 2007). The blockade of these two cytokines is proved to ameliorate the inflammation (Yamamoto et al., 2000; Coccia et al., 2012). Our results validated that FMT could downregulate these two cytokines, leading to the amelioration of inflammation reflected by the reduction of CRP. It has been reported that loss of VDR in intestinal epithelial or myeloid cells will facilitate mucosal pro-inflammatory cytokine expression and exacerbate experimental colitis (Leyssens et al., 2017). In concert with this study, the elevated level of VDR in this study is attributable to the protection of intestinal mucosa by FMT from the inflammatory injury. Additionally, the upregulated expression of IL-10 suggests an efficacious inflammatory inhibition in intestinal mucosa of the responsive group patients. Taken together, FMT could attenuate inflammation by inhibiting the expression of pro-inflammatory cytokines and augment the expression of VDR.

High taxonomic and metabolic observed OTUs and Shannon index seemed to be positively associated with more severe inflammation (high full Mayo score and pro-inflammatory cytokines levels), despite that CRP negatively correlated with the Shannon index. The genera which have significant coefficients with clinical biomarkers are the most pro-inflammatory genus and have been reported to be linked with UC previously (Tang et al., 2021; Xu et al., 2021; Zhuang et al., 2021). Collinsella has been reported to efficiently colonize in the solid mucin-agar part of mucosal surfaces and belong to pro-inflammatory bacteria (Astbury et al., 2020; van Soest et al., 2020). However, there has been one study that showed specific Collinsella strain could produce butyric acid (Qin et al., 2019), which might explain why it is correlated with improvement of clinical index.

The comparison of responsive and nonresponsive patients showed the shift of microbiota only in the responsive group. Accordingly, there are no differential taxonomic or metabolic features after FMT identified in nonresponsive patients. It is postulated that the failure of microbiota transplantation is responsible for the poor clinical efficacy in the nonresponsive group. In the responsive group, the decreased enterobacteria, which is generally considered to play a pro-inflammatory role in UC, is the most likely cause of inflammation reduction. As expected, the previously reported altered taxa associated with UC were also found in our study, including the enrichment of Proteobacteria, Enterococcus, and Turicibacter, and also the lack of Anaerostipes, Coprococcus, Roseburia, Faecalibacterium, Ruminococcus, and Gemmiger (Tang et al., 2021; Volkova and Ruggles, 2021; Xu et al., 2021; Zhuang et al., 2021). There was no differential taxa observed in the nonresponsive group at week 6 and we speculated this might be attributed to the slight change and small sample size. Patients in our study exhibited reduced amino acids synthesis and increased degradation, reduced short chain fatty acid production, higher bacterial antigen synthesis, and higher vitamin K synthesis, which were reported to be associated with CDI but not UC (Nowak et al., 2014). FMT could correct a majority of these alterations. Liposaccharides and heme production is higher in nonresponsive patients and have been reported to be associated with failure of clinical response (Paramsothy et al., 2019), although in our study their enrichment was observed before treatment. Decreased fatty acids seem to contribute to the success of response which still needs further validation. The long-term examination of gut microbiota revealed a rebound of taxa and metabolic functions 1 year after FMT. There has been a report about how the application of additional nutrient supplementation following FMT could enhance the efficacy of FMT for metabolic diseases (Mocanu et al., 2021). We hope the addition of amino acids and short chain fatty acid might also help to improve the efficacy of FMT for UC treatment. The two-way ANOVA failed to prove significant effect of response on all measurements above, suggesting the key factor associated with clinical response still needs to be identified in future studies.

As for oral microbiota, we intended to explore if the amelioration of UC in the gut after FMT could lead to the reduction of oral symptoms and the alteration of oral microbiota. We also wanted to identify specific oral bacteria, which have a correlation with clinical index as in previous studies (Said et al., 2014; Xun et al., 2018). Due to the small sample size, the results regarding oral microbiota are only to provide reference for related studies and also need to be cross-validated. In this study, the fecal microbiota were processed under strictly anaerobic conditions since the oxygen would affect the most anti-inflammatory bacteria. The gut microbiota of children have been reported to possess higher abundance of Bifidobacterium and Faecalibacterium and lower abundance of Bacteroides (Hollister et al., 2015), and it also showed trends toward enrichment of functions correlated with anti-inflammatory properties, which all are associated with better health condition (Le Chatelier et al., 2013). The optimal route for FMT to treat UC is still uncertain and we chose a route according to the Montreal classification of extent. The relatively higher clinical response and remission rates in this study, compared with other studies, reported in a systematic review conducted by Costello et al. (2017) proved all these procedures together contribute to the amelioration of UC.

Our study has several limitations. Firstly, we did not have a placebo group and the sample size is not large. Secondly, only part of the oral microbiota, blood, and intestinal mucosa were sampled. Third, the metabolic alterations were inferred *in silico* and need further validation by other means. The following study will expand the sample size and set up a randomized control.

In conclusion, FMT using fecal microbiota from young donors seems to be an effective and safe approach for active UC treatment. FMT could efficiently downregulate levels of pro-inflammatory cytokines and inflammation biomarkers. A successful shift of microbiota composition is crucial for clinical responsiveness after FMT therapy. The efficacy of FMT could last for more than 1 year in spite of a tendency of microbiota to rebound. These findings may not only provide a valuable reference for pathogenesis study but also help improve the therapeutic strategy for ulcerative colitis.

## Data Availability Statement

The datasets presented in this study can be found in online repositories. The names of the repository/repositories and accession number(s) can be found in the article/supplementary material.

## Ethics Statement

The studies involving human participants were reviewed and approved by the ethics committee of The First Affiliated Hospital of Anhui Medical University (No. PJ2018-03-07). The patients/participants provided their written informed consent to participate in this study. Written informed consent was obtained from the individual(s) and minor(s)’ legal guardian/next of kin, for the publication of any potentially identifiable images or data included in this article.

## Author Contributions

XC and B-LS conceived and designed the trial. W-HZ collected data. Z-YJ, Z-HY, and J-YZ performed bioinformatics and statistical analysis. X-HM and JG drafted the manuscript. W-HZ and Z-YJ edited the manuscript. All authors contributed to the article and approved the submitted version.

## Funding

This work was supported by grants from the Key Research and Development Plan Project of Anhui Province, Department of Science and Technology (201904a07020043).

## Conflict of Interest

The authors declare that the research was conducted in the absence of any commercial or financial relationships that could be construed as a potential conflict of interest.

## Publisher’s Note

All claims expressed in this article are solely those of the authors and do not necessarily represent those of their affiliated organizations, or those of the publisher, the editors and the reviewers. Any product that may be evaluated in this article, or claim that may be made by its manufacturer, is not guaranteed or endorsed by the publisher.

## Footnotes

^1^ https://github.com/GalaxyRyuk/QIIME2/blob/master/qiime2_pipeline.sh
